# Expression of Inhibin-Alpha Is Regulated Synergistically by Wilms' Tumor Gene 1 (Wt1) and Steroidogenic Factor-1 (Sf1) in Sertoli Cells

**DOI:** 10.1371/journal.pone.0053140

**Published:** 2013-01-11

**Authors:** Shao-Yang Ji, Jian-Xiu Hao, Lei Li, Jun Zhang, Qiao-Song Zheng, Xi-Xia Li, Xiao-Na Wang, Chun-Sheng Han, Fei Gao, Yi-Xun Liu

**Affiliations:** 1 State Key Laboratory of Reproductive Biology, Institute of Zoology, Chinese Academy of Sciences, Beijing, China; 2 Graduate School of the Chinese Academy of Sciences, Beijing, China; Leibniz Institute for Age Research - Fritz Lipmann Institute (FLI), Germany

## Abstract

*Wt1* encodes a zinc finger nuclear transcriptional factor, which is specifically expressed in testicular Sertoli cells and knockdown of Wt1 in Sertoli cells causes male mice subfertility. However, the underlying mechanism is still unclear. In this study, we found that expression of inhibin-α is significantly reduced in *Wt1*-deficient Sertoli cells. Luciferase assays using the inhibin-α promoter indicated that the inhibin-α promoter is transactivated by the Wt1 A, and B isoforms (−KTS), but not the C, and D isoforms (+KTS). Analysis of the Wt1 responsive element of the inhibin-α promoter region using site-directed mutagenesis showed that the nucleotides between −58 and −49 are essential for Wt1-dependent transactivation of the inhibin-α promoter. ChIP assays indicated that Wt1 directly interacts with the inhibin-α promoter. In addition, the inhibin-α promoter is activated synergistically by Wt1 and Sf1. Mutation of the ligand binding domain (LBD) of Sf1 (residues 235–238) completely abolished the synergistic action between Wt1 and Sf1, but did not affect the physical interaction between these two proteins, suggesting that other factor(s) may also be involved in the regulation of inhibin-α in Sertoli cells. Further studies demonstrated that β-catenin enhances the synergistic activation of Wt1 and Sf1 on the inhibin-α promoter. Given the fact that inhibin-α, a subunit of inhibin, is known to be involved in the regulation of spermatogenesis and testicular steroidogenesis, this study reveals a new regulatory mechanism of inhibin-α in Sertoli cells and also sheds light on the physiological functions of Wt1 in gonad development and spermatogenesis.

## Introduction

Inhibins were first isolated and identified in gonadal fluids and extracts based on their ability to inhibit the synthesis and secretion of follicle-stimulating hormone (FSH) in the pituitary gland [1]. Inhibins are composed of two subunits, a common α subunit and one of two β subunits, A and B, encoded by separate genes [2], [3]. Inhibin-α is expressed predominantly in ovarian granulosa cells and testicular Sertoli cells and has structural homology with transforming growth factor (TGF-β) [4]–[7]. It has been reported that the expression of inhibin-α in granulosa cells is regulated by orphan nuclear receptor steroidogenic factor 1 (Sf1) [8], [9], and cAMP [9]. The Wnt signaling pathway [10] is also involved in the regulation of inhibin-α expression by synergistically acting with Sf1.

The *Wt1* gene encodes a nuclear transcription factor which is predominantly expressed in kidneys, gonads, the spleen, and mesothelium during embryo development [11]. In adults, Wt1 expression is restricted to kidney podocytes, ovarian granulosa cells, and testicular Sertoli cells [12]. Wt1 protein contains a proline/glutamine-rich N-terminal transcriptional regulatory domain and has a DNA binding domain with four C2-H2 zinc fingers in its C-terminus [13], [14]. A number of growth and differentiation related genes, such as insulin-like growth factor II [15], [16], insulin-like growth factor I receptor [17], platelet-derived growth factor A-chain [18], [19], Pax-2 [20], syndecan-1 [21], Dax-1 [22], and amphiregulin [11], [23], [24], are regulated by Wt1.

Inactivation of *Wt1* in testicular Sertoli cells between E12.5-E14.5 results in testicular dysgenesis [25]. Knockdown of Wt1 by siRNA in mature Sertoli cells causes male subfertility [26], suggesting that Wt1 plays a role in testicular development and spermatogenesis. However, its underlying mechanism is still unknown. To investigate the mechanism of Wt1 in testicular development and spermatogenesis, we used real-time PCR to examine genes expressed differentially between control and *Wt1*-deficient Sertoli cells. We found that inhibin-α was significantly reduced in *Wt1*-deficient Sertoli cells. Further analysis revealed a putative Wt1 binding sequence in the 5′-flanking DNA sequences of the inhibin-α gene, suggesting that Wtl protein binds to the promoter and functions as an activator of the inhibin-α gene in Sertoli cells *in vivo*.

## Materials and Methods

### Plasmid Construction

Mouse *Wt1* cDNA was amplified by PCR using testicular cDNA and subcloned into a pCMV-Tag2b vector. The primers used were as follows: 5′-TAC TGG ATC CGG TTC CGA CGT GCG GGA C-3′ and 5′-TCA GAA TTC TGC CTG GGA TGC TGG AC-3′. The Mouse inhibin-alpha promoter with different lengths (−745, −79, −53, +11, and +40 to +171) were amplified by PCR and subcloned into a pGL3-basic luciferase reporter vector (Promega Corp., Madison, WI), and named −745, −79, −53, +11, +40 inhibin-α Luc (Inh-α Luc), respectively. The primers used for amplifying the inhibin-α promoter were: 5′-CTA GCC TCT TTA CCC TGG ACC CT-3′, 5′-GCT AGC CTT CCC CCA CAT TC-3′, 5′-GCT AGC GGA GAT AAG GC-3′, 5′-GCT AGC GGG CGA CTG GGA C-3′, 5′-GCT AGC GAC TGG GGG AGA C-3′, and 5′-TTC GAA AGT TCA CTT GCC CTG ATG-3′. The Wt1REM-Inh-α Luc (consensus sequence deletion) reporter vector was generated using a QuikChange® II Site-Directed Mutagenesis Kit according to the manufacturer's instructions. Primers used for generating the Wt1RE mutant were: 5′- CCC CTT CCC CCA CAT TCT TGG AGA TAA GGC TCA GG -3′ and 5′- CCT GAG CCT TAT CTC CAA GAA TGT GGG GGA AGG GG -3′. The inhibin-α Sf1RE (−42 to −34) mutant construct was generated by introducing a point mutation into the Sf1 binding site by PCR and was named Sf1REM Inh-α Luc. The oligonucleotides used for generating the Sf1RE mutant were: 5′- GTG GGA GAT AAG GCT CAG TTC CAC AGA CAT CTG CG-3′ and 5′-CGC AGA TGT CTG TGG AAC TGA GCC TTA TCT CCC AC-3′. An HA-tagged Sf1 expression vector [9] was generously provided by J. Larry Jameson (Northwestern University, Chicago, IL). The pcDNA3-S37A-β-catenin was generously provided by Wancai Yang (University of Illinois at Chicago, Chicago, IL). The Sf1(235-4A) vector was generated using the method described above with the following oligonucleotides: 5′-GCT GCT GCA ACT AGC GGC AGC GGC GGA CCA GGT GCG CG-3′, and 5′-CGC GCA CCT GGT CCG CCG CTG CCG CTA GTT GCA GCA GC-3′. All plasmids were sequenced before use.

### Cell culture and transfection

The Sertoli cell-derived cell line, TM4 [27], was cultured in DMEM/F12 medium supplemented with 5% horse serum, 2.5% fetal bovine serum, 100 µg/ml streptomycin and 100 IU/ml penicillin, at 37°C in 5% CO_2_. Cells were trypsinized and plated at a density of 7×10^4^ cells/well in a 24-well culture plate overnight before transfection. TM4 cells were transfected with 300 ng of luciferase reporter plasmids, 10 ng of phRL-TK and 100 ng of other expression plasmids using FUGENE HD transfection reagent (Roche Applied Science, Mannheim, Germany) according to the manufacturer's directions. Cells were harvested 36 h after transfection for luciferase assays, and 40 h for co-immunoprecipitation and Western Blotting or ChIP assays.

### Luciferase Reporter Assays

TM4 cells were transiently co-transfected with a constant amount of pGL3-Inhibin-α (−745) or other mutant luciferase reporter constructs (300 ng), and pRL-TK Renilla luciferase reporter plasmid (10 ng) with or without 100 ng of the expression plasmids for Flag-Wt1, HA-Sf1, Sf1 (235–238 4A), S37A-β-catenin or an empty plasmid. The total amount of plasmid DNA for each transfection was kept constant. Cells were harvested and lysed 36 h after transfection, and both firefly and Renilla luciferase activity was measured using a dual luciferase reporter assay system (Promega). The firefly luminescence signal was normalized based on the Renilla luminescence signal.

### ChIP Assays

Chromatin immunoprecipitation (ChIP) assays were performed according to a protocol provided by Upstate Biotechnology (Charlottesville, VA). In brief, TM4 cells were transiently transfected with the Flag-Wt1 expression plasmid. Cells were crosslinked 48 h after transfection with 1% formaldehyde. Cells were then washed in ice-cold phosphate-buffered saline (PBS) and resuspended in 200 µl of SDS lysis buffer containing a protease inhibitor mixture. The suspension was sonicated on ice and pre-cleared with protein A-agarose beads blocked with sonicated salmon sperm DNA (Upstate Biotechnology) for 30 min at 4°C. Beads were removed, and the chromatin solution was immunoprecipitated with polyclonal anti-Flag (Sigma) antibody and normal mouse IgG (Santa Cruz) at 4°C, followed by incubation with protein A-agarose beads for an additional 1 h at 4°C. Immune complexes were eluted with 100 µl of elution buffer (1% SDS and 0.1 M NaHCO_3_), and formaldehyde cross-links were reversed by heating at 65°C for 6 h. Proteinase K was added to the reaction mixtures and incubated at 45°C for 1 h. DNA from the immunoprecipitates and control input DNA were purified and then analyzed by standard PCR. PCR amplification of the proximal inhibin-α promoter region spanning the Wt1 response element was performed using the following primers: 5 ′- GGG GTG GTG CAT TCT GTC CT- 3′ and 5 ′-GCT GCC CTG TGC CCT TTC TGT-3′; and the distal region of inhibin-α promoter (−7.5 to −7 kb) was performed using the following primers: 5 ′-GGA CCC CCA CCA AGC CAA CAG AC-3′ and 5 ′-TCA GCC CTA CAC CAG CAC GCA GAC-3′ [10].

### Real-time Quantitative PCR

Total RNA was extracted from TM4 cells using TRIzol reagent (Invitrogen) according to the manufacturer's instructions, and reverse transcription was performed with SuperScript II reverse transcriptase (Invitrogen). Real-time quantitative PCR (TaqMan PCR) was carried out using an ABI Prism 7700 sequence detection system (Perkin-Elmer Applied Biosystems, Foster City, CA) according to the manufacturer's protocol. The PCR primers used are as follows: Wt1, 5′-CCA GTG TAA AAC TTG TCA GCG AAA -3′, 5′-ATG AGT CCT GGT GTG GGT CTT C-3′; inhibin-α, 5′-GGC CAT CCC AAC ACA TAC G-3′, 5′-ACC AAG GTG TGT CCG GAT CC-3′; GAPDH, 5′-TGA TGA CAT CAA GAA GGT GGT GAA G-3′, 5′-TCC TTG GAG GCC ATG TAG GCC AT-3′. All reactions were performed in triplicate. mRNA levels for each of the genes were normalized against GAPDH.

### Co-immunoprecipitation and Western Blotting

TM4 cells were transiently transfected with 1 µg of Flag-Wt1 A expression vector (or empty pCMV-tag2b) and 1 µg of HA-Sf1 expression vector or HA-Sf1(235-4A) using FUGENE HD. Cells were harvested 48 h after transfection and washed with ice-cold phosphate-buffered saline before being disrupted with hypotonic lysis buffer (50 mM Tris-HCl, pH 8.0, 150 mM NaCl, 0.5% Nonidet P-40, 5 mM EDTA) supplemented with protease inhibitors. Cell debris was removed by centrifugation after incubation for 30 min at 4°C. About 0.8 mg of protein extract was incubated with 20 µl of anti-Flag-M2 affinity gel (Sigma) or normal mouse IgG (Santa Cruz) at 4°C for 4 h. Immunoprecipitated proteins were washed three times with lysis buffer and then analyzed by Western blotting.

### Isolation of primary Sertoli cells

All animal work was approved by the committee on animal care at the Institute of Zoology, Chinese Academy. The Permit Number is TEL2010050016. Testes from 3-week-old mice were decapsulated under a dissection microscope. Seminiferous tubules were pooled and washed three times with PBS. Tubules were then incubated with 2 mg/ml collagenase I (Sigma) and 0.5 mg/ml DNase I (Sigma) in DMEM for 30 min at 37°C on a shaker, washed twice with DMEM and further digested with 2 mg/ml collagenase I, 0.5 mg/ml DNase I and 1 mg/ml hyaluronidase type III (Sigma) for 20–30 min at 37°C on a shaker. The tubules were allowed to settle and were then washed twice with DMEM before further digesting with 2 mg/ml collagenase I, 0.5 mg/ml DNase I, 2 mg/ml hyaluronidase, and 1 mg/ml trypsin for 40–60 min at 37°C on a shaker. This final digestion step resulted in a cell suspension primarily containing Sertoli cells and type A spermatogonia. The dispersed cells were then washed twice with DMEM and placed into culture dishes in DMEM containing 10% fetal calf serum and incubated at 37°C (5% CO2). Spermatogonia were unable to attach to the dish and were removed after changing the medium on the following day. 4OH-Tamoxifen (Sigma, H7904) was dissolved in ethanol to generate a 1 mM stock solution and was further diluted to appropriate concentrations prior to use. Recombination was initiated by adding 4OH-TM to cultured Sertoli cells at a final concentration of 1 µm. Total RNA was extracted from cells after 3 days of culture as described previously.

## Results

### The expression of inhibin-α is significantly reduced in Wt1-deficient Sertoli cells

**Figure 1:**
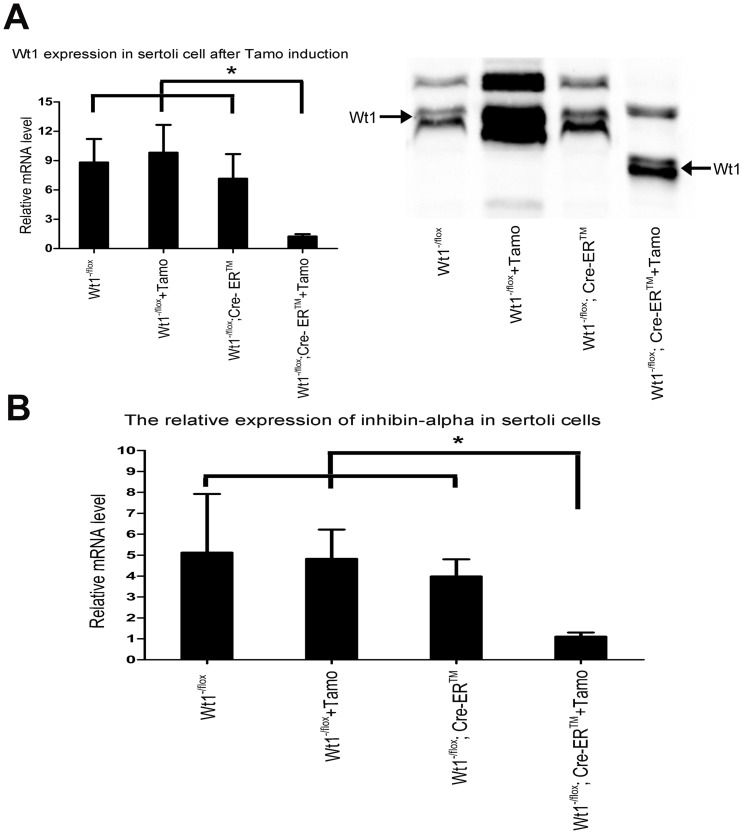
Expression of Inhibin-α was significantly reduced in Wt1-deficient Sertoli cells. Wt1 was inactivated in Sertoli cells by Tamoxifen induction. The expression of *Wt1* and *Inhibin-α* was examined by Real-time PCR and Western blot. A, *Wt1* expression was dramatically reduced in *Wt1^−/flox^; Cre-ER™* sertolic cells after tamoxifen treatment. B, Compared to controls, the expression of inhibin-α was significantly decreased in Tamoxifen treated *Wt1^−/flox^; Cre-ER™* Sertoli cells.

Our earlier work indicated that Wt1 is essential for testicular development [25], however, the underlying mechanism of Wt1 action is still unclear. To identify putative Wt1 target genes in Sertoli cells, we generated *Wt1^−/flox^; Cre-ER™* mice by crossing *Wt1^+/−^; Cre-ER™* males with *Wt1^flox/flox^* females. Primary Sertoli cells were isolated from the testes of 3-week-old mice and Wt1 was inactivated by Tamoxifen induction. The expression of *Wt1* and inhibin-α was significantly reduced in *Wt1^+/−^; Cre-ER™* Sertoli cells after Tamoxifen induction (Fig. 1A,B), while the expression of *Wt1* and inhibin-α in Tamoxifen-treated control Sertoli cells did not change, indicating that the decrease of inhibin-α in *Wt1^+/−^; Cre-ER™* Sertoli cells was not due to the side effects of drug treatment. These results indicate that inhibin-α is one of *Wt1* downstream genes.

### The inhibin-α promoter is transactivated by Wt1 A and B isoforms (-KTS)

**Figure 2:**
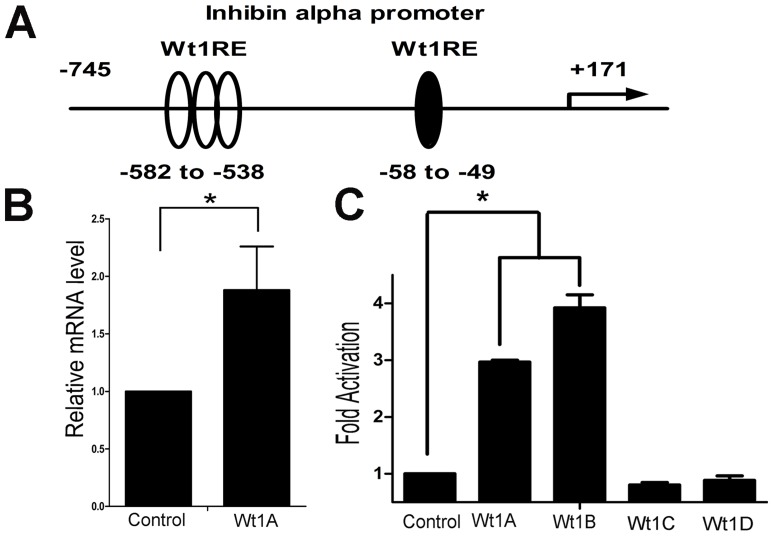
The inhibin alpha promoter is transactivated by the Wt1 A and B isoforms (−KTS), but not the C and D isoforms (+KTS). A, Schematic drawing of inhibin-α promoter. The open and filled ovals indicate the putative Wt1 binding sites. B, Real-time PCR results showed the elevated endogenous inhibin-α expression in primary Sertoli cells after transfection with Wt1A. C, The inhibin-α promoter was transactivated by the Wt1 A and B isoforms (−KTS), but not the C and D isoforms (+KTS). TM4 cells were transiently transfected with a luciferase reporter plasmid termed pGL3-Inhibin alpha (−745) and a Renilla luciferase reporter plasmid along with Wt1-expressing vectors. Luciferase activity was examined 36 h later. Compared to control cells, the luciferase activity was significantly increased in Wt1 A and B transfected TM4 cell, not in C and D isoforms transfected cells. (* p<0.05, Wt1A: −Exon5, −KTS; Wt1B: +Exon5, −KTS; Wt1C: −Exon5, +KTS; Wt1D: +Exon5, +KTS).

Luciferase assays were performed to investigate the regulatory mechanism of Wt1 in the expression of inhibin-α. Based on previous studies [8], [9], we generated a luciferase reporter vector bearing the mouse inhibin-α promoter spanning from positions −745 to +171, named Inh-α Luc (−745), four putative Wt1 binding sites were identified in the inhibin-α promoter region (Fig. 2A). To examine the possible contribution of Wt1 to inhibin-α gene expression, TM4 cells were transiently transfected with a constant amount of Inh-α Luc (−745), and Renilla luciferase reporter plasmid together with an empty plasmid or a plasmid expressing vectors of different Wt1 isoforms. Cells were lysed 36 h after transfection, and luciferase activity was measured. The luciferase activity of Wt1 A and B (−KTS) transfected TM4 cells increased 3–4 fold compared to control cells (Fig. 2C). In contrast, no difference was noted between control and Wt1 C and D (+KTS) transfected TM4 cells. These results further confirm that the expression of inhbin-α in Sertoli cells is regulated by Wt1. To further confirm this result, the primary Sertoli cells were transfected with Wt1 A. As shown in Fig. 2B, expression of endogenous inhibin-α was significantly increased compared to control cells.

### Identification of a Wt1-responsive element within the inhibin-α promoter

**Figure 3:**
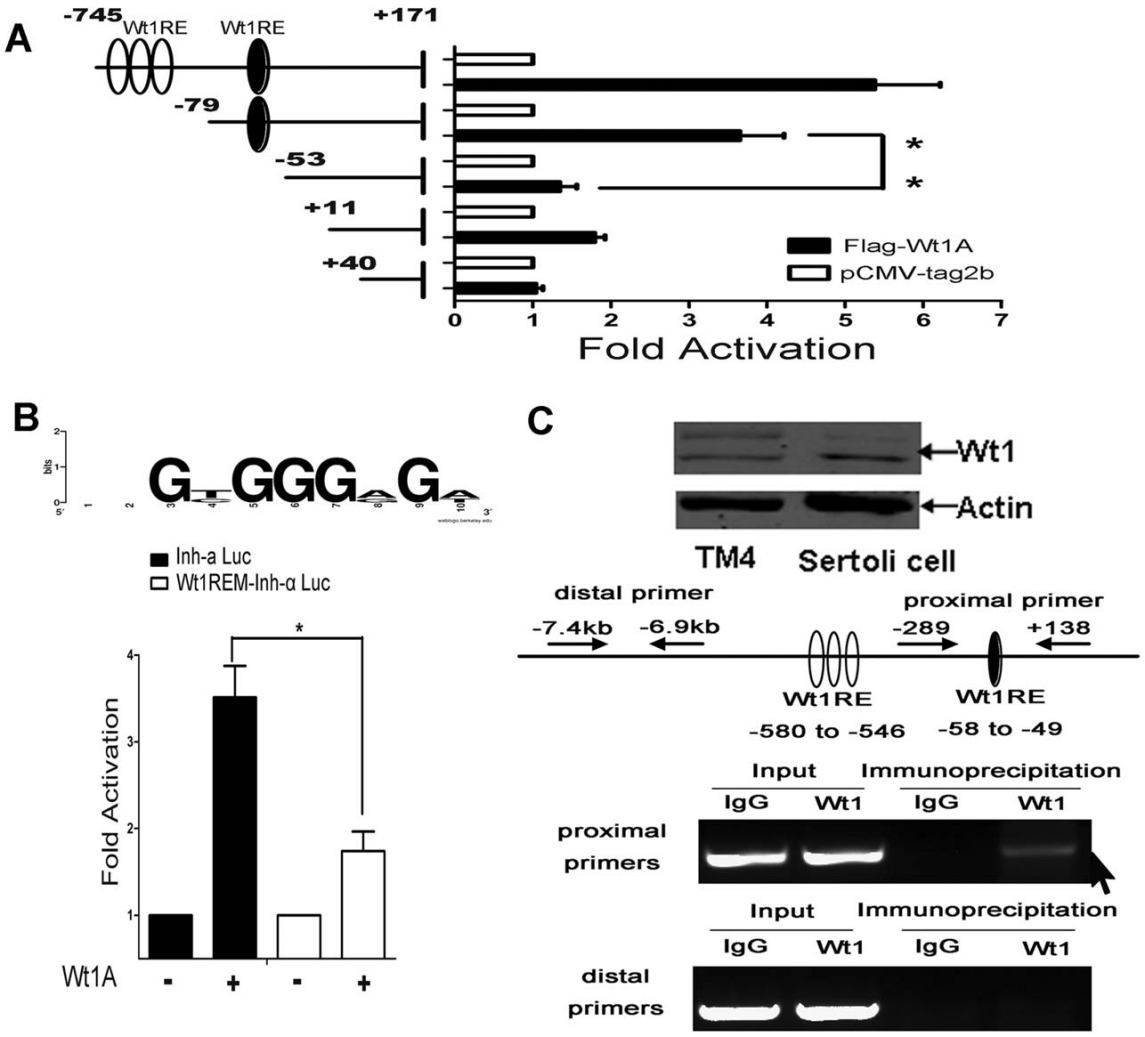
Identification of the DNA sequence within the mouse inhibin-α promoter required for Wt1A-dependent transcriptional activation. A, TM4 cells were transiently transfected with luciferase reporter constructs bearing different lengths of the mouse Inhibin alpha promoter sequence, and Renilla luciferase reporter plasmids together with an empty plasmid (pCMV-tag2b) or Wt1A expression vector. The filled and open ovals indicated putative Wt1 response elements (Wt1RE). B, The Wt1-dependent transactivational activity of inhibin-α promoter was significantly decreased after deletion of the ten base pairs (5′- GGC GGG AGT G -3′) sequence including part of the Wt1 consensus binding motif. C, Western blot showed the endogenous Wt1 protein expression in TM4 cells and primary Sertoli cells. TM4 cells were transiently transfected with the empty plasmid or with the expression plasmid for Flag-Wt1A. 48 h after transfection, cells were cross-linked with formaldehyde and cross-linked chromatin was sonicated followed by immunoprecipitation with anti-Flag antibody. Genomic DNA was purified from the immunoprecipitates and subjected to PCR using the primers as indicated in schematic drawing of Inhibin-α promoter and distal primers as negative control. A specific PCR product (black arrow) was amplified in Flag-Wt1A transfected cells with proximal primers, not with the distal primers. Normal mouse IgG served as a negative control.

To identify the region within the inhibin-α promoter that is essential for Wt1-dependent transcriptional activation, we generated a series of progressively 5′ truncated inhibin-α promoter reporter constructs and assayed their luciferase activity. Luciferase activity was unchanged with promoter deletions up to −79 (Fig. 3A), however it decreased dramatically compared to the control group with promoter deletions up to −53, indicating that there is a Wt1 responsive element between −79 and −53 of the inhibin-α promoter. ChIP assays were conducted to examine whether Wt1 can be recruited to this essential region of the inhibin-α promoter. For this purpose, two sets of primers: the proximal primers (−289, and +138) and the distal primers (−7.5 to −7 kb) were designed to amplify the desired region of the inhibin-α promoter (Fig. 3C). We examined the endogenous Wt1 protein expression in TM4 cell line using Western blot, as shown, the protein level of Wt1 in TM4 cells was lower than primary Sertoli cells (Fig. 3C). TM4 cells were transiently transfected with empty or Wt1-Flag expressing plasmids. Genomic DNA extending from −289 to +138 (427 bp) was specifically amplified (Fig. 3C), but no specific DNA band was amplified in control cells, and no specific band was amplified with distal primers in both *Wt1* overexpressed and control cells, suggesting that the Wt1 protein interacted with the promoter region of the inhibin-α gene.

Having shown that a Wt1 consensus binding motif (5′- GAG TGG GAG A -3′) is present between −58 and −49 (Fig. 3C (filled ovals) and Fig. 3B (LoGo)), we addressed the functional significance of this motif in the regulation of Wt1-dependent transcriptional enhancement of inhibin-α. Luciferase assays were performed with using a luciferase reporter vector bearing a mutant inhibin-α promoter in which the ten base pairs (5′- GGC GGG AGT G -3′) sequence including part of the Wt1 consensus binding motif was deleted, termed Wt1REM-Inh-α Luc (consensus sequence deletion). Results indicated that deletion of this sequence dramatically reduced (from about 3.5∼folds to 1.7∼folds) luciferase activity mediated by Wt1 (Fig. 3B), suggesting that this sequence may act as a Wt1-responsive element for inhibin-α gene expression.

### The inhibin-α promoter is synergistically activated by Wt1 and Sf1

**Figure 4:**
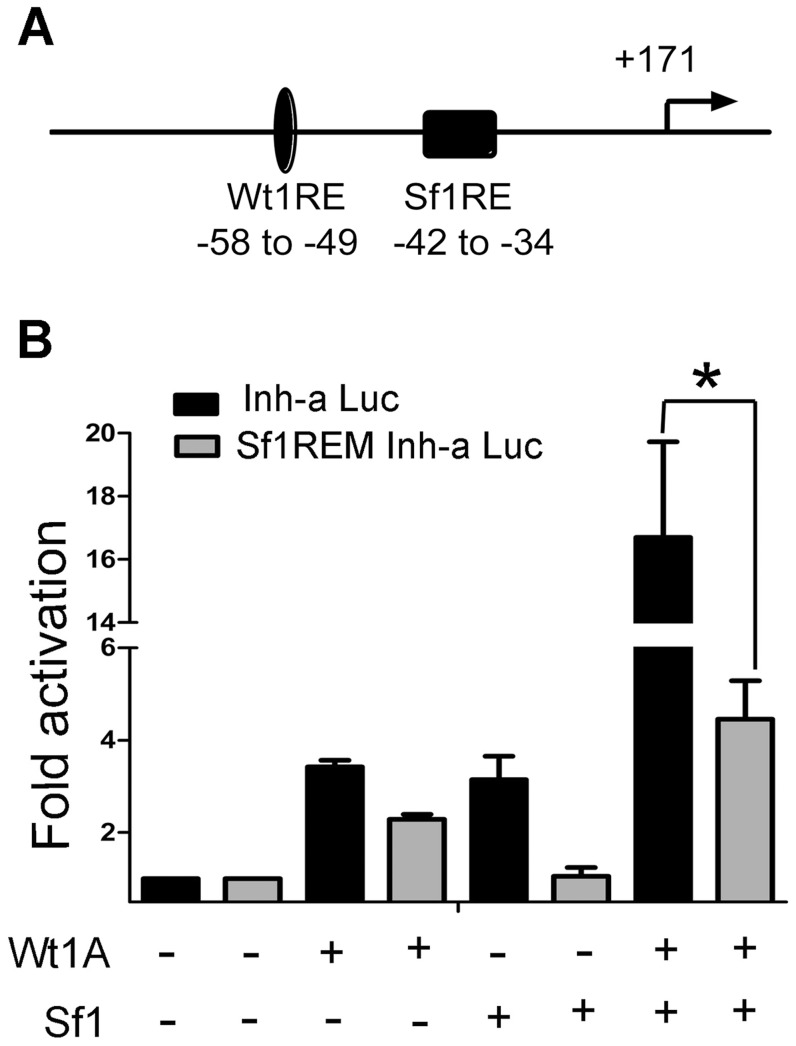
The inhibin-α promoter is synergistically activated by Sf1 and Wt1. A, Schematic drawing of the inhibin-α promoter region. The filled oval and box indicate the putative Wt1 and Sf1 responsive elements. B, Co-transfection of Wt1 and Sf1 significantly activated the inhibin-α promoter compared to Wt1 or Sf1 alone; luciferase activity increased about 4-fold. In contrast, when the inhibin-α promoter luciferase reporter vector with a mutated Sf1 responsive element sequence (5′-TCA GGG CCA-3′ to 5′- TCA GTT CCA-3′), Sf1REM Inh-α Luc, was co-transfected with Wt1 and Sf1, the synergistic action between Wt1 and Sf1 was completely abolished.

**Figure 5:**
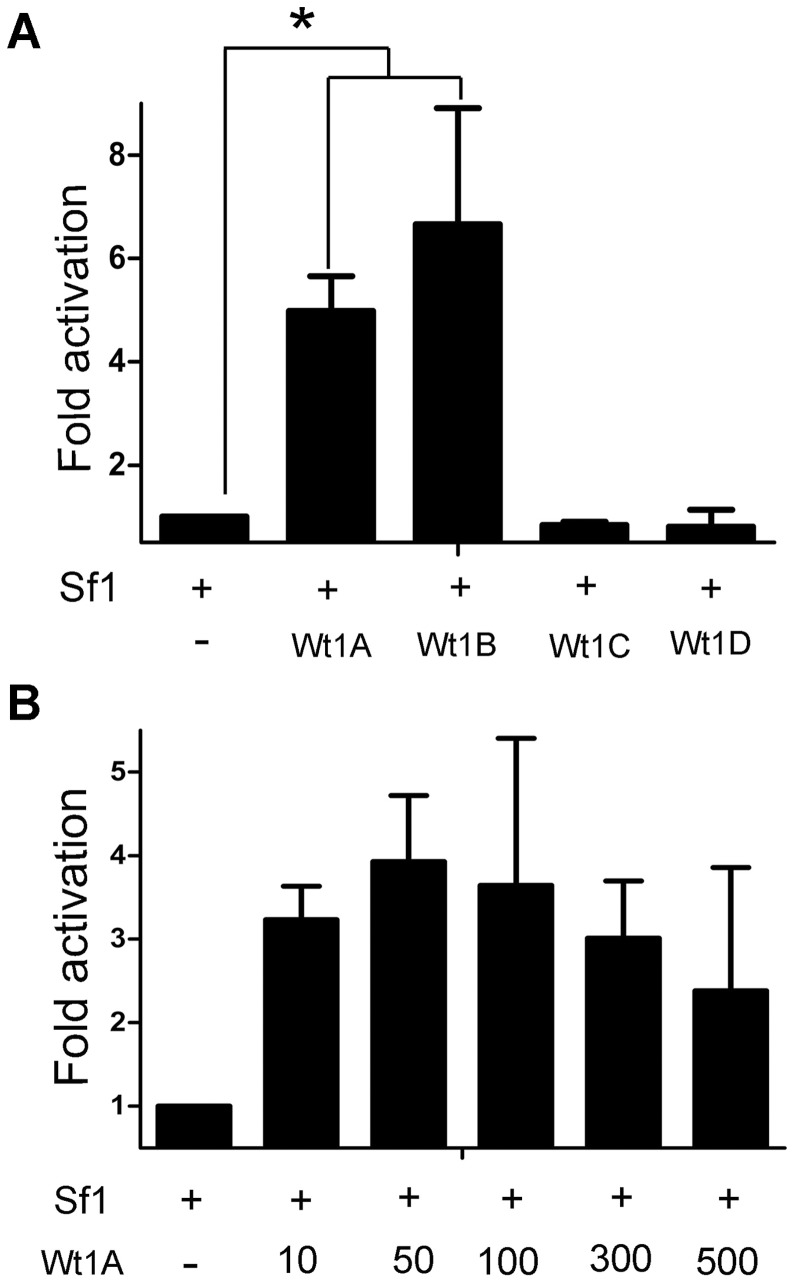
The inhibin-α promoter is synergistically activated by Sf1 and the Wt1 A and B, but not C and D isosforms, and Wt1 synergizes with Sf1 in a dose-independent manner. A. Luciferase activity indicated that the inhibin-α promoter is significantly transactivated by Wt1 A and B and Sf1 co-transfection, but transfection of Sf1 and Wt1 C and D isoforms did not change the luciferase activity compared to the control. B. No difference in luciferase activity was noted when Sf1 was co-transfected with different doses of Wt1 A (10, 50, 100, 300, 500 ng).

Sf1 is a transcriptional regulator of steroidogenic enzyme genes [28], [29], and is expressed in testicular Sertoli cells. Previous studies have shown that Sf1 plays an essential role in inhibin-α gene expression [8], [9]. A conserved Sf1-responsive element (5′-TCA GGG CCA-3′) was found within the inhibin-α promoter adjacent to the Wt1 binding site (Fig. 4A), suggesting possible synergistic activation of Wt1 and Sf1 in inhibin-α expression. To address this question, TM4 cells were transfected with Sf1, Wt1, and an inhibin-α luciferase reporter plasmid. As shown in Fig. 5A, luciferase activity in Wt1A/B(−KTS) and Sf1 co-transfected cells was 3–4 fold higher than that in cells transfected with Wt1 or Sf1 alone. However, Sf1 and Wt1 C/D (+KTS) co-transfection did not affect luciferase activity. To further confirm this result, we generated an inihibin-α luciferase reporter bearing a mutant Sf1-responsive element (TCA GGG CCA→TCA GTT CCA), termed Sf1REM Inh-α Luc (−745). Luciferase assay results indicated that the synergistic activation of the inhibin-α gene by Wt1 and Sf1 was completely abolished (Fig. 4B). We also found that inhibin-α promter was transactivated with Wt1 with a dose indenpendent manner, no difference in luciferase activity was noted when Sf1 was co-transfected with different doses of Wt1 A (10, 50, 100, 300, 500 ng) (Fig. 5B).

### Residues 235–238 in the Sf1 Ligand-Binding Domain (LBD) are essential for the synergistic action of Wt1 and Sf1

**Figure 6:**
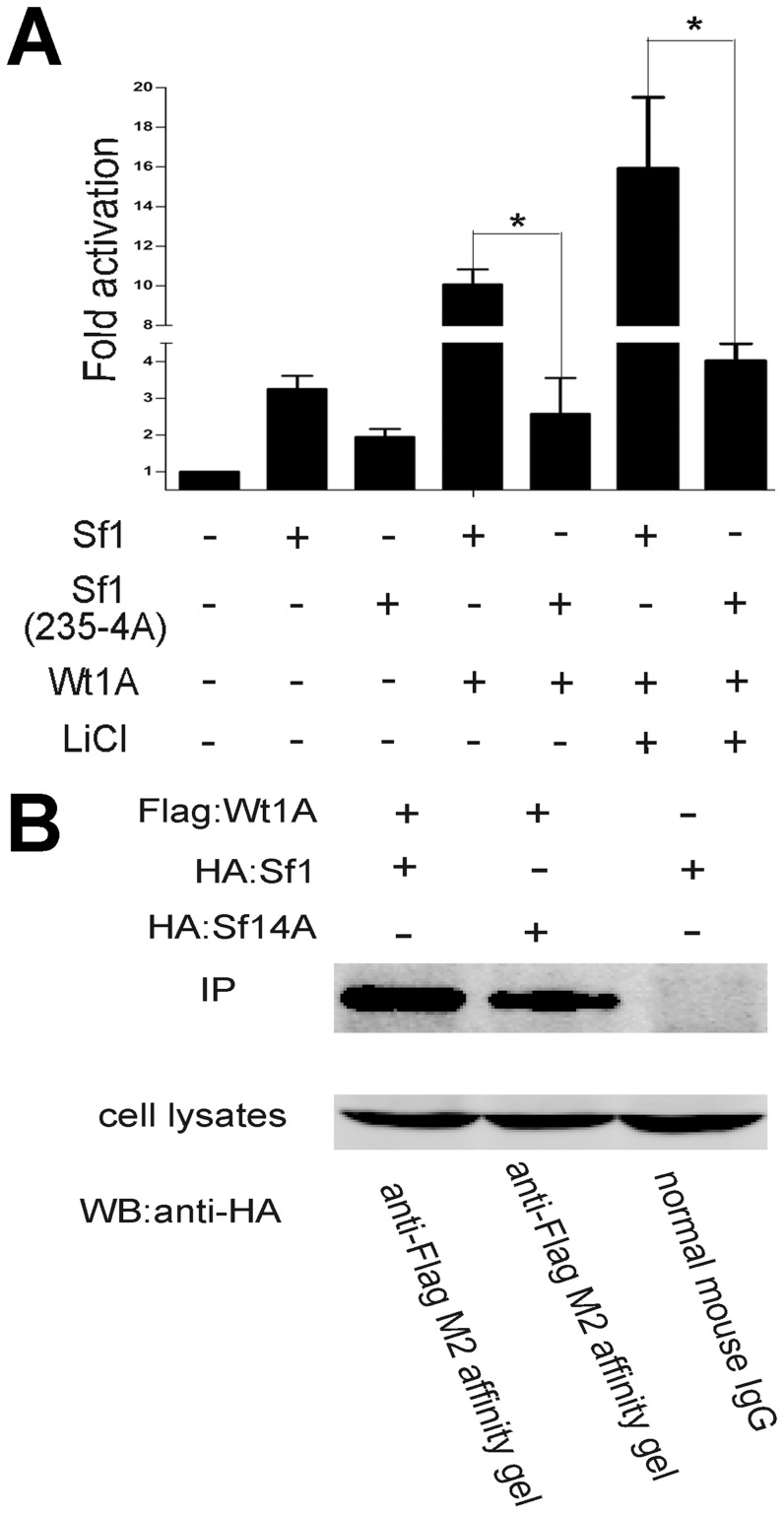
The Sf1 ligand-binding domain (residues 235–238) is essential for Wt1 and Sf1 synergistic activation of the inhibin-α promoter, but does not affect the interaction between Wt1 and Sf1. A. Mutations of the Sf1 ligand-binding domain (residues 235–238) completely abolish the synergistic action of Wt1 and Sf1 on the inhibin-α promoter. B. Interactions between Wt1 and Sf1 proteins were assessed by co-immunoprecipitation; both wild type Sf1 and Sf1 (235–238 4A) interacted with Wt1 protein. Normal mouse IgG served as a negative control.

Amino acids 235–238 in the Sf1 Ligand-Binding Domain are conserved in all vertebrate species [30]. It has been reported that these four residues in Sf1 are essential for interaction with β-Catenin [30], [31], and that the inhibin-α promoter is synergistically activated by Sf1 and β-Catenin [8]. To test the functional significance of these four Sf1 residues on the synergistic action of Wt1 and Sf1 on inhibin-α expression, Sf1 residues 235–238 were substituted by four alanines. We found that mutating the LBD of Sf1 completely eliminated the synergistic activation of Wt1 and Sf1 on the inihibin-α promoter (Fig. 6A). Physical interaction between Wt1 and Sf1 has previously been confirmed using pull-down assays [32]. We performed co-immunoprecipitation to test whether the four Sf1 amino acids are critical for the interaction between Wt1 and Sf1. Both Sf1 and Sf1(235-4A) were pulled down by the anti-Flag M2 affinity gel (Fig. 6B), suggesting that Sf1 amino acids 235–238 are essential for its functional interaction with Wt1 to synergistically transactivate the inhibin-α promoter.

### The synergistic action of Wt1 and Sf1 on the inhibin-α promoter is enhanced by Wnt signaling

**Figure 7:**
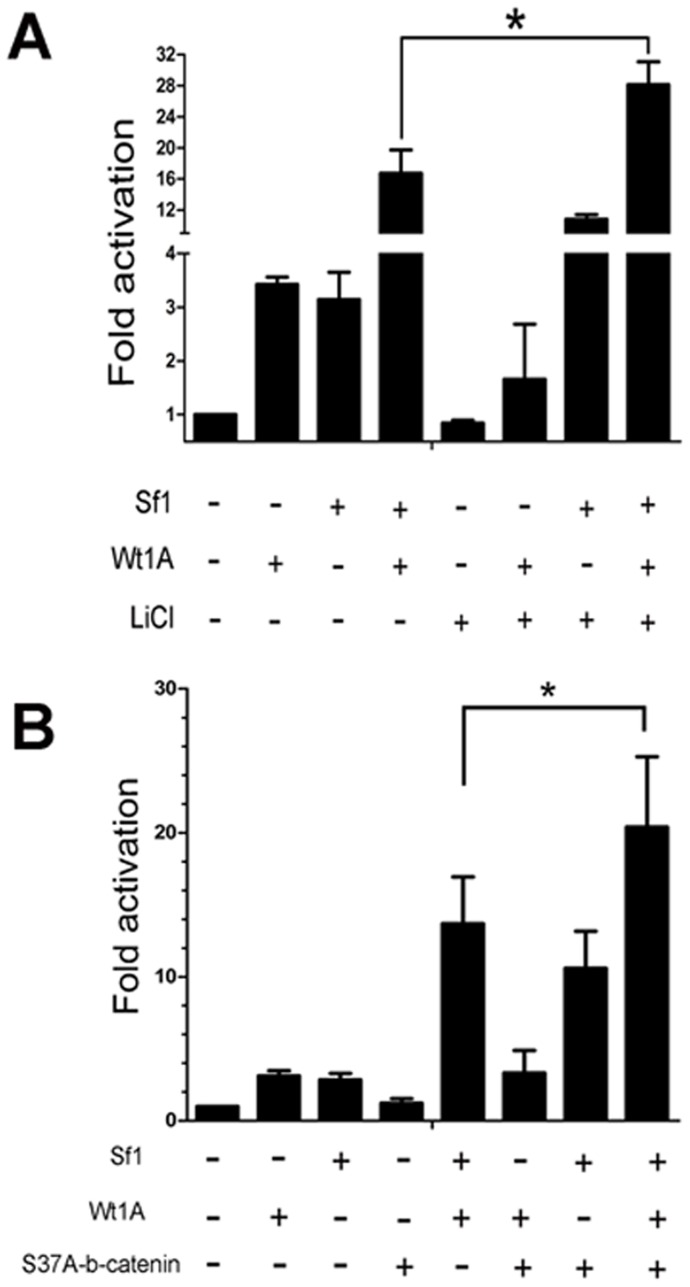
Synergistic activation of the inhibin-α promoter by Wt1 and Sf1 is enhanced by Wnt signaling, but the inhibin-α promoter is not synergistically activated by Wt1 and Wnt signaling. A, Compared to Wt1 and Sf1 co-transfected TM4 cells, co-transfection of constitutively activated β-catenin, S37A could significantly induced the luciferase activity. In contrast, S37A β-catenin alone did not promote the activity of inhibin-α promoter induced by Wt1 only. B, The same results were obtained when TM4 cells were treated with 40 mM LiCl treatment.

It has previously been reported that Wnt signaling is involved in Sf1-induced inhibin-α expression [8]. In this study, Wnt signaling pathway was activated by LiCl treatment or transfection with constitutive active β-catenin (S37A). We found that Sf1 and Wnt signaling synergistically activate the inhibin-α promoter (Fig. 7 A,B). In contrast, no synergy between Wt1 and Wnt signaling on the activation of the inhibin-α promoter was observed (Fig. 7 A,B). Intriguingly, the activity of the inhibin-α promoter was significantly induced in TM4 cells when Wt1 and Sf1 were co-transfected along with LiCl treatment or β-catenin (S37A) transfection, suggesting that Wnt signaling enhances Wt1 and Sf1 induced inhibin-α expression in Sertoli cells.

## Discussion

Inhibins, inhibitors of follicle-stimulating hormone (FSH) synthesis and secretion in the pituitary gland, are predominantly produced in males by Sertoli cells. The physiological functions of inhibins in testicular development and spermatogenesis have been studied using *in vivo* models. Overexpression of the α subunit of inhibin in mice causes a reduction in testis size and sperm production [33]. In contrast, knockout of the inhibin-α gene leads to elevated FSH levels and stromal tumor development in testes, in turn causing progressive testicular damage [34]. These studies indicate that inhibins are involved in the regulation of testicular development and spermatogenesis.

Wt1, a transcriptional factor, is specifically expressed in testicular Sertoli cells. Previous reports have indicated that Wt1 may play a role in spermatogenesis [26]. But the exact function of *Wt1* in spermatogenesis is still largely unknown. In this study, we found that the expression of inhibin-α was dramatically decreased in *Wt1-*deficient Sertoli cells, indicating that inhibin-α is a potential target gene of *Wt1* and that the expression of inhibin-α is induced by *Wt1* in Sertoli cells. However, in granulosa cells the expression of inhibin-α is repressed by Wt1 [8], and the expression of FSHR is regulated by Inhibins through paracrine [35], suggesting that *Wt1* function is probably cell-type dependent. Although the phenotype of *Wt1* and inhibin-α knockout mice is different in testes development, given the fact that inhibin-α plays important roles in spermatogenesis, we speculated that *Wt1* involved in spermatogenesis was partially mediated by inhibin-α. But we could not exclude the possibility that other genes were also regulated by *Wt1*.

We further demonstrated that the inhibin-α promoter is transactivated by Wt1 A and B isoforms (−KTS), but not the C and D isoforms (+KTS), consistent with previous studies. The Wt1 (−KTS) isoforms modulate transcriptional activity of reporter constructs [36]–[38]. In contrast, the Wt1 (+KTS) isoforms are involved in pre-mRNA splicing [39].

Four potential Wt1 consensus binding sites (5′- GAG TGG GAG A -3′) were found within the inhibin-α promoter, three of which were located between −580 and −546, and one between −58 and −49. However, assays of truncated reporters indicated that only the sequence between −58 and −49 is essential for Wt1-dependent inhibin-α promoter activation. This was further confirmed by site-directed mutagenesis (Fig. 3B); deletion of this sequence within the inhibin-α promoter completely abolished Wt1-dependent inhibin-α promoter activation. ChIP assays suggested that Wt1 binds directly to this region and that this sequence element may act as a Wt1-responsive element for the murine inhibin-α gene.

Sf1 was originally identified as a transcriptional regulator of steroidogenic enzyme gene expression [40]. Expression of Sf1 is restricted to endocrine tissues, such as the gonads, adrenal cortex, ventromedial hypothalamus, and the pituitary gland [41]. Previous studies have indicated that Sf1 is involved in the regulation of inhibin-α expression. In the present study, we have demonstrated for the first time that Sf1 acts synergistically with Wt1 to activate the inhibin-α promoter. We also found that mutation of four residues in the LBD of Sf1 completely eliminates the synergistic action of Wt1 and Sf1 on the inhibin-α promoter, but does not change the interaction of these two proteins, suggesting that the interaction between Wt1 and Sf1 is not mediated by the LBD in Sf1. It is possible that endogenous factors may interact with Sf1 via the LBD to enhance the synergistic transactivation of Wt1 and Sf1. It has been reported that Sf1 interacts with β-catenin through the LBD to recruit other factors to stimulate the inhibin-α promoter [8], [9], [30]. We speculated that β-catenin is probably also involved in the synergistic action of Wt1 and Sf1 on inhibin-α expression by interacting with the LBD of Sf1. To test this hypothesis, the Wnt pathway was activated by LiCl treatment or co-transfection of constitutive activeβ-catenin (S37A). Luciferase assays showed that the inhibin-α promoter was not synergistically activated by Wt1 and Wnt signaling, but β-catenin (S37A) transfection or LiCl treatment significantly enhanced the synergistic action of Wt1 and Sf1, suggesting that the Wnt signaling pathway is involved in the regulation of inhibin-α expression in Sertoli cells by promoting the synergistic action of Wt1 and Sf1.

In summary, we have demonstrated for the first time that the expression of inhibin-α is transactivated by *Wt1* in Sertoli cells. Nucleotides between −58 and −49 are essential for Wt1 binding to the inhibin-α promoter. In addition, the expression of inhibin-α is synergistically induced by Wt1 and Sf1, and this synergy is promoted by Wnt signaling, probably by interacting with the LBD of Sf1. This study reveals a new regulatory mechanism of inhibin-α in Sertoli cells and also sheds light on the physiological functions of *Wt1* in gonad development and spermatogenesis.
